# The African Field Epidemiology Network-Networking for effective field epidemiology capacity building and service delivery

**Published:** 2011-12-14

**Authors:** Sheba Nakacubo Gitta, David Mukanga, Rebecca Babirye, Melissa Dahlke, Mufuta Tshimanga, Peter Nsubuga

**Affiliations:** 1 African Field Epidemiology Network, Kampala, Uganda; 2 Zimbabwe Field Epidemiology Training Program, Department of Community Medicine, Harare, Zimbabwe; 3 Center for Global Health, Centers for Disease Control and Prevention, Atlanta, USA

**Keywords:** field epidemiology, network, African Field Epidemiology Network, public health workforce

## Abstract

Networks are a catalyst for promoting common goals and objectives of their membership. Public Health networks in Africa are crucial, because of the severe resource limitations that nations face in dealing with priority public health problems. For a long time, networks have existed on the continent and globally, but many of these are disease-specific with a narrow scope. The African Field Epidemiology Network (AFENET) is a public health network established in 2005 as a non-profit networking alliance of Field Epidemiology and Laboratory Training Programs (FELTPs) and Field Epidemiology Training Programs (FETPs) in Africa. AFENET is dedicated to helping ministries of health in Africa build strong, effective and sustainable programs and capacity to improve public health systems by partnering with global public health experts. The Network's goal is to strengthen field epidemiology and public health laboratory capacity to contribute effectively to addressing epidemics and other major public health problems in Africa. AFENET currently networks 12 FELTPs and FETPs in sub-Saharan Africa with operations in 20 countries. AFENET has a unique tripartite working relationship with government technocrats from human health and animal sectors, academicians from partner universities, and development partners, presenting the Network with a distinct vantage point. Through the Network, African nations are making strides in strengthening their health systems. Members are able to: leverage resources to support field epidemiology and public health laboratory training and service delivery notably in the area of outbreak investigation and response as well as disease surveillance; by-pass government bureaucracies that often hinder and frustrate development partners; and consolidate efforts of different partners channelled through the FELTPs by networking graduates through alumni associations and calling on them to offer technical support in various public health capacities as the need arises. AFENET presents a bridging platform between governments and the private sector, allowing for continuity of health interventions at the national and regional level while offering free exit and entry for existing and new partners respectively. AFENET has established itself as a versatile networking model that is highly responsive to members’ needs. Based on the successes recorded in AFENET's first 5 years, we envision that the Network's membership will continue to expand as new training programs are established. The lessons learned will be useful in initiating new programs and building sustainability frameworks for FETPs and FELTPs in Africa. AFENET will continue to play a role in coordinating, advocacy, and building capacity for epidemic disease preparedness and response.

## Introduction

Whereas advances in science and technology have resulted in the development of many life-saving and affordable technologies [1], many developing countries have poor health indices and are unlikely to attain the targets for the health-related Millennium Development Goals (MDGs) and other internationally agreed goals [2]. Sub-Saharan Africa registers the highest global disease burden at 24% despite accounting for only 11% of the world's population [3].The World Health Organisation (WHO) attributes the poor health indicators to weak health systems and has proposed a health systems framework to counter this problem. The WHO framework consists of six essential system building blocks: service delivery; health workforce; information; medical products, vaccines and technology; financing; and, leadership/governance [4]. An adequate health workforce is central to all the other building blocks.

Of the 57 countries faced with an extreme shortage of health workers, 36 are in Africa [3]. African and Asian countries have an estimated shortfall of 4.2 million health workers [5]. Laboratories are the weakest link in Africa's public health system, affecting timely confirmation and response to disease outbreaks as well as proper diagnosis and clinical care of patients [6].

Despite the marked increase in financial resources made available to low-income countries in the 21^st^ century, many are still grappling with weak health systems. Some have attributed this grim picture to the fact that most of the aid coming to the health sector is earmarked for specific programs leaving some critical areas unfunded and other areas over funded. For example, HIV/AIDS accounts for 30% of the disease burden in low-income countries and receives 46% of donor funding, compared to acute respiratory infections which account for 26% of the disease burden but receive < 2.5% of direct funding [7]. Faced with competing demands, a high disease burden, and limited resources, low-income countries need to devise mechanisms to accomplish much more with the limited resources.

One approach to that has been advocated to maximally use the limited resources is to develop and use partnerships and networks that advance knowledge and technology sharing.

## What is networking?

Several definitions of networks exist. In general, a network is a relational organizational form consisting of individuals, groups or organizations that come together, interact and share resources to pursue a common goal in a coordinated and concerted way [8,9]. If the network is made up of individuals or groups such as teams, divisions, or regional sites within the same organization, it is an intra-organizational network, while if it is made up of organizations then it is an inter-organizational network [10].Inter-organization networks may be classified into three categories based on the governance type namely: shared governance, lead organization governed, and network administrative organization governed [11].

Members of a network share goals and processes that extend beyond two organizations to a multitude of organizations that create a unified response to a given phenomenon [12,13].The key incentive for participation in a network is the opportunity to achieve a goal that otherwise each member would not be able to achieve alone. There is a trade-off between the long-term collective benefits of the networks such as innovation and new product development, community wellbeing, buffering against environmental (e.g., political, economic, market, social) turbulence and the short-term individual pay offs of participating organizations such as attainment of recognition and legitimacy. Participating organizations must have something to add as well as takeaway from the network. Network members retain benefits of remaining formally independent and small while reaping economies of scope and scale through the structure of other organizations [14]. Members have flexibility in terms of resources of time and cost [15,16], an organization is dependent upon others in its environment to bestow legitimacy and recognition upon it.

## Why public health networks?

Partnerships and networking are proven approaches to addressing public health challenges. Networks help leverage scarce resources to attain a common goal. For example, WHO's Global Laboratory Network, strengthens laboratory support to immunization programs. These laboratory networks include: measles and rubella, poliomyelitis, yellow fever, and human papillomavirus (HPV) networks [17]. Other examples of existing networks are the Global Outbreak Alert and Response Network (GOARN) [18]; the Global Influenza Network ( i.e., FLU-LAB);the World Bank-funded East African Public Health Laboratory Network (EAPHLN),whose focus is on strengthening laboratories to improve access to diagnostic services for tuberculosis in the African region; and the Partners in Population and Development Regional Office's three regional networks which promote integrated reproductive health programs in Africa: the East African Reproductive Health Network (EARHN), the Western African Reproductive Network (WARHN), and the Southern Africa Reproductive Health Network (SARHN) [19].

The Training Programs in Epidemiology for Public Health Interventions Network (TEPHINET) is a global network of field epidemiology and allied training programs that was created in 1997 to provide support, peer review, and quality assurance for its member programs [20]. In 2004, field epidemiology training programs in Africa, recognizing that there were areas of need particular to Africa that could be addressed by a regional network, began discussions to setup a network that would address their unique needs and challenges [21].These programs now constitute the AFENET founding members [22].

Despite having several partnerships and networks in the public health arena in sub-Saharan Africa, a majority are disease specific or narrow in scope, addressing only a few components of the health system. Many of the partnerships are North-South collaborations with very few South-South collaborations. We describe an inter-organizational network established in 2005 by Field Epidemiology Training Programs (FETPs) and a Field Epidemiology and Laboratory Training Program (FELTP) in Africa [23,24]. The African Field Epidemiology Network (AFENET) is a hybrid of a shared and network administration organization governance network [11]. Member programs work collectively to make strategic and operational decisions under the framework of a General Assembly and a Board of Directors, while a Secretariat under leadership of an Executive Director performs the day to day running of the network, offering both technical and administrative support under guidance of the latter [22]. The General Assembly is the senior governance body and is constituted by all members of AFENET. It elects the eight-member Board of Directors whose composition consists of representatives from the Network (two founder members, one ordinary member, and two member countries), a private sector representative, an eminent member of society with vast public health experience, and a representative from WHO Regional Office for Africa as a non-voting member.

## The African Field Epidemiology Network(AFENET)

The African Field Epidemiology Network (AFENET) was established in 2005 as a not-for-profit regional organization and networking alliance of African FETPs and FELTPs, dedicated to helping ministries of health in Africa build strong, effective, and sustainable programs with the capacity to improve public health systems by partnering with global public health experts. The Network's goal is to strengthen field epidemiology and public health laboratory capacity to contribute effectively to addressing epidemics and other major public health problems in Africa. AFENET currently networks 12 FETPs and FELTPs in sub-Saharan Africa with operations in 20 countries. The Network's Secretariat is based in Kampala, Uganda. AFENET's operations are guided by six strategic priorities described in Table 1.

**Table 1:** African Field Epidemiology Network (AFENET)'s strategic priorities

Strategic Priorities	Indicator
Field epidemiology capacity development	Critical mass of well-trained field epidemiologists ensuring effective prevention and control of epidemics and other major public health problems.
Public health laboratory capacity development	Well-equipped, staffed, linked and functional public health laboratory infrastructure.
Public health disease surveillance and effective response	Early detection, timely and effective response and reduced mortality from epidemics and other major public health problems in Africa.
Networking and collaboration	Field epidemiologists and laboratories effectively engaged in value-adding collaboration and partnerships.
AFENET's institutional development	AFENET demonstrates good management systems and processes.
Documentation and publication	Regular dissemination of the network's activities in relevant scientific media and the establishment of effective information storage and retrieval mechanisms.

AFENET Secretariat, under the leadership of an Executive Director, has a team of financial, technical and administrative staff organised under four units: Technical Programs, Science and Public Affairs, Finance, and Administration.

AFENET's operations are funded by several implementing partners (www.afenet.net). One of these is the United States (U.S.) Centers for Disease Control and Prevention (CDC) which provided the seed funding that has supported AFENET's operations since inception. As a CDC principal implementing partner for FELTPs in sub-Saharan Africa [25], AFENET receives substantial funding from CDC through cooperative agreements to support member programs as well cover networking operational costs. This mechanism enables CDC to play an active role in the implementation of FELTPs through the provision of technical assistance in addition to financial support.

### What gaps does AFENET address?

Field Epidemiology and Laboratory Training Programs (FELTPs) offer a 2-year competency-based training in applied epidemiology and public health laboratory practice. Trainees acquire an array of practical public health skills in disease surveillance and outbreak investigation, data management, monitoring and evaluation of health programs, scientific writing and communication. Approximately 70% of their time is spent in the field, with trainees learning by doing and in the process contributing to public health service delivery in liaison with local health teams.

AFENET supports networking of FELTP trainees by sponsoring their travel to both regional and international scientific meetings and conferences using funds from implementing partners. These meetings provide trainees with opportunities to disseminate their study findings with the wider public health audience, including policy makers, while at the same time interacting with trainees from other programs and public health practitioners.

The establishment of a FELTP is a multi-stage process, the first of which is usually an initial country assessment to assess the feasibility of establishment of a FELTP [25]. AFENET supports African countries to establish FELTPs by participating in the initial country assessment and advocacy activities. Once the program is established, AFENET usually participates in recruitment of personnel; offers administrative, financial and logistical support; resource mobilization; and participates in teaching and other program activities. Logistics management is challenging in many African countries, given the lengthy bureaucratic procedures instituted by governments. AFENET provides an alternate platform that bypasses these procedures and has proved to be a valuable mechanism especially during outbreaks.

Resource mobilization through several approaches is a critical function that AFENET offers its membership. Field Epidemiology Training Programs are a proven approach to addressing public health challenges, but are more resource-intensive than traditional public health training programs because of the field component [20]. AFENET's business development unit, which is responsible for grant writing and management, plays a key role in this process. To date, AFENET has been awarded more than 10 grants (a majority being CDC cooperative agreements, and with major support from the U.S. Agency for International Development (USAID)) to support FELTPs and ministries of health in epidemiology training, responding to outbreaks, strengthening public health surveillance, and public health laboratory capacity. Other methods applied to mobilize resource include advocacy and networking with existing and potential partners.

Over the years, AFENET has widened its international partner base to include other U.S. government agencies, U.S. Foundations, U.S. organizations, WHO, GOARN, the European Centres for Disease Control and Prevention (ECDC), and the European Union. With this support, AFENET continues to work towards strengthening and expanding field epidemiology and laboratory capacity across Africa.

AFENET plays an advocacy role for field epidemiology capacity building in Africa. This is achieved through meetings with potential funders, government officials and academia in countries that have expressed interest in establishing FELTPs, dissemination of reports from the various member FELTPs through newsletters, annual reports and publications, and presentations at meetings/conferences. The acute shortage of health workers in Africa is aggravated by “brain drain” of its health workers. Available data show that 40% of graduates from medical and allied health schools migrate to other countries [26, 27]. It has been reported elsewhere that 85% of graduates from African FELTPs stay and work in their home countries for at least 3 years after completing training [24]. This is very encouraging since FELTPs train a select few every year through the 2-year program, hence it is critical to retain graduates in country or on the continent. In addition to the 2-year training program, FETPs and FELTPs provide short term in-service training for front-line health workers. Graduates from both programs contribute towards bridging the human resource gap in their respective countries.

AFENET maintains a database of African FELTP graduates using Epi-Track, a customized MS AccessTMdatabase, which was developed in close collaboration with CDC. In the recent past, AFENET has been championing the establishment of alumni associations for its member programs as a networking and tracking strategy. The Zimbabwe program launched its alumni association in September 2008. Since then, other programs have followed suit. The Kenya FELTP Alumni Association, with support from AFENET, conducted a grant writing workshop in July 2010. In March 2011, AFENET supported the Uganda program to hold a two-day symposium for its alumni under the theme of ‘One Health’, during which a Uganda FETP Alumni Association governing committee was elected into office. The Kenya and Uganda alumni meetings were funded by CDC and USAID's Emerging Pandemic Threats Program respectively. The Nigeria FELTP also has an active alumni association.

The FELTP graduates database is a valuable pool of human resources that can be called upon by AFENET and its members to address public health challenges in any of its member countries and Africa as a whole. To date, 489 trainees have graduated from African FELTPs. These graduates are available to the WHO Regional Office for Africa (WHO AFRO), GOARN and others responding to infectious disease outbreaks and other health emergencies.

The pool of graduates is augmented by public health experts from member programs and partners; this expertise is shared amongst the membership, for example, as guest lecturers and external examiners for review of trainees’ theses.

The public health systems and quality assurance committee of the AFENET Board of Directors is charged with the responsibility of assuring quality of the member FELTPs. Since 2010, annual assessments have been conducted across the various programs and certificates are awarded indicating the programs’ level of performance. The committee recommends continuous quality improvement activities which are evaluated periodically.

AFENET membership presents a common platform for addressing African public health issues since it has representation from Anglophone(Ethiopia, Ghana, Kenya, Nigeria, Rwanda, South Africa, South Sudan, Tanzania, Uganda and Zimbabwe), Francophone (Burkina Faso, Cameroon, Central Africa Republic, Democratic Republic of Congo, Mali, Niger, Togo) as well as Lusophone (i.e., Portuguese speaking) countries, namely, Angola and Mozambique. FELTPs within a particular language zone therefore have opportunities to provide support to each other in areas of information sharing and teaching expertise.

The Network allows for implementation of multi-country programs and projects. AFENET already has a platform and mechanism to implement projects across countries. It is able to overcome government bureaucracies that often hinder and frustrate development partners through its strong linkage with ministries of health across the network. This provides value addition to donors interested in multi-country work, while members enjoy participation.

In line with AFENET's key priority of public health disease surveillance and effective response, African FETPs and FELTPs continue to play a critical role in the roll out the WHO's Integrated Disease Surveillance and Response (IDSR) in Africa [24]. AFENET is also actively involved in International Health Regulations (IHR, 2005) [28], especially cross border issues especially amongst its membership.

### Examples of AFENET's success stories

AFENET plays an instrumental role in helping membership countries investigate and respond to outbreaks in a timely manner. First, through funding from CDC (provided by USAID), AFENET secretariat has funds earmarked for outbreak support which it is able to make available to an affected member country within 48 hours of notification of a disease outbreak through its financial infrastructure across the membership. This allows for timely investigation and response to outbreaks while at the same time providing FELTP trainees with opportunities to practice their newly-acquired skills. Table 2 summarises some of the outbreaks that occurred in the past 5 years that have been investigated by AFENET member programs and highlights the role played by trainees.

**Table 2:** Summary of outbreaks investigations conducted in the African Field Epidemiology Network (AFENET) member programs

Program	Outbreaks (Year)	Trainees’ involvement
**Central Africa**	Polio (2011), cholera (2011)	Data collection and analysis, summaries of epidemiological information
**Ethiopia**	Measles, whooping cough, rabies, anthrax, acute watery diarrhoea, polio, malaria, malnutrition, river pollution (2010)	Determined factors associated with disease (measles), health education (malaria, malnutrition)
**Ghana**	Measles, H1N1, (2010, 2011), Influenza A (2007, 2009), food-borne outbreaks (2010), rabies (2010), whooping cough (2010), meningitis (2007, 2011)	Identified magnitude, source, factors contributing to outbreak and implement preventable and control measures, Influenza: contact follow-up
**Kenya** *	Cholera (2008, 2009), influenza A, rift valley fever (2006-2007), aflatoxicosis (2007), nodding disease (2010), Leishmaniasis (2010), Meningitis (2007), Rabies (2011), yellow fever (2011)	Surveillance and investigation, *Vibrio Cholera* isolation
**Mozambique**	Measles, cholera, food-borne pesticide intoxication	Case identification, line listing, source investigation
**Nigeria**	Meningitis (2007, 2009), lead poisoning (2010), cholera (2009, 2010), polio (2011), cerebro-spinal meningitis (2011), RVF (2007), flurosis (2011), Lassa fever (2009, 2011), avian Influenza (2007), Di-ethylene glycol poisoning (2009), leptospirosis	Assess magnitude of outbreak, identify risk factors, provide response activities, health education, case management, lab confirmation (cholera). laboratory confirmation, training of health care workers on the use of personal protective equipment, and community health education(Lassa fever), Identify risk factors, hygiene and sanitation, health education and rodent management (leptospirosis)
	Lead –poisoning	
**Rwanda**	Cholera (2009, 2010), botulism (2009), food poisoning (2009), food poisoning (2009)	Case definition, line listing, case identification, community sensitization
**South Africa**	Rift valley fever (2010), E. Coli, *Pseudomonas aeruginosa*, H1N1 in ostriches	Sero-survey of exposed vets (H1N1)
**Tanzania**	Rift Valley Fever(2007), measles (2010), cholera (2010, 2011), viral haemorrhagic-fever, 2011, bomb blasts (2011),	Immunisation campaigns, index case tracing, health education
**Uganda**	Marburg/Ebola (2007, 2008), Ebola (2011), cholera (2007), anthrax (2007), hepatitis E (2008), malaria (2009, 2010), polio (2010), Yellow fever (2011), dysentery, influenza, plague (2007), meningitis (2007)	Screening, case identification, surveillance, training health workers, training community health workers, health education, sensitization (Ebola), Health capacity to assess disease, community sensitization (anthrax), case identification (hepatitis E), Response interventions, health education, treatment interventions (malaria), Identify causative agent and source of outbreak, Health education (dysentery), Case management, surveillance, laboratory support, contact tracing (H1N1), spraying fleas (plague)
**West Africa**	Cholera, meningitis, yellow fever, measles (2010)	Index case tracing, line listing, health education, vaccination campaigns (measles)
**Zimbabwe**	Cholera (2008), anthrax (2008, 2009), malaria (2008), rabies (2008), salmonellosis, dysentery (2010), typhoid (2010), measles (2010)	Source and case identification, control measures, health education, improvement of sanitation, Case finding, education campaigns (anthrax), Characterise outbreak, determine factors associated with disease (measles)

* The Kenya FELTP trains residents from South Sudan

Second, AFENET with the support of CDC procures and assembles outbreak investigation laboratory kits which it supplies to its membership [29]. These kits are customised to meet individual country needs such as provision of anti-sera laboratory kits for commonly occurring pathogens. The kit contents include: personal protective equipment, rapid diagnostic tests or point-of-care diagnostics, specimen collection bottles, and transportation media. Through bulk purchasing, the Network enjoys economies of scale and scope. The rapid diagnostic tests help ensure timely confirmation of disease outbreaks and allow for better case management to minimise morbidity and case fatalities.

AFENET's support to Zimbabwe during the 2008-2009 cholera epidemic that affected the entire country is an excellent case study of benefits that accrue from membership to a network. In December 2008 and January 2009, the Zimbabwe Ministry of Health and Child Welfare received support from AFENET to assist in the control of the cholera epidemic. The support was provided through the Zimbabwe FETP and included oral rehydration salts, gumboots, facemasks, aprons, surgical gloves and laboratory antimicrobial sensitivity testing discs. The donations from AFENET were procured using CDC funds and focused on protecting the frontline health workers and saving lives of cases [29].

Third, the network structure allows for easy sharing of information and resources across the network and with partners. Of particular interest is shipment of laboratory specimens across geographical borders during outbreaks to reference laboratories in Africa and outside the continent. During the 2006-2007 Rift Valley Fever (RVF) outbreak that affected Tanzania and Kenya [30–31], information sharing and technical collaborations between the respective FELTPs in the two neighbouring countries contributed towards timely investigation and containment of the outbreak. Graduates from the Kenya FELTP participated in the 2008 Ebola outbreak investigations in Uganda [32–33]. Through assisting Ugandan counterparts to contain the outbreak, the Kenya public health system's capacity to respond to Ebola and other viral haemorrhagic fevers was strengthened.

The growing network and partnerships that have been built create opportunities for sharing scientific research activities and findings. Through the regional AFENET conferences held biennially, trainees from the various programs have presented scientific abstracts based on activities undertaken during their training. The last two AFENET Regional conferences were held in Uganda and Kenya in 2007 and 2009, respectively, and the next conference will be held in Tanzania in December 2011.

Beginning in 2008, AFENET, through its partnership with the Pan African Medical Journal (PAMJ), hosts the editorial office for this journal with the aim of encouraging scientific publication among its membership. Several trainees, alumni and staff from AFENET's member programs have published in the journal on topics varying from pesticide poisoning to HIV status disclosure among women attending PMTCT in Zimbabwe. Three scientific writing workshops have been held to support trainees to develop manuscripts for publication. Starting with the 2011 AFENET Scientific Conference, PAMJ-AFENET scientific writing workshops will be held as part of the pre-conference activities at all AFENET scientific conferences. This journal presents the Network with a forum to share scientific outputs with the wider scientific audience.

In 2009, AFENET collaborated with the Nigeria FELTP, the U.S. Department of Health and Human Services’ Office of Global Health Affairs, and Usmanu Danfodiyo University to establish a training program for lower- and mid-level health workers, referred to as “The Health Diplomacy Program”. The program has trained 87 health staff in basic epidemiological skills and health leadership and management skills. This initiative has helped strengthen the public health workforce by not only focusing on the top-tier level of management but also focusing on the lower tiers that form the bulk of the health work force.

A number of other types of training programs have been initiated due to networking among partners. For example, AFENET, through the Ghana FELTP, adapted CDC's Sustainable Management Development Program (SMDP), which supports public health leaders and managers by enhancing their management capacity, to the African context and developed the Ghana Management Training Centre. Courses such as Health Management and Information Systems (HMIS) and Improving Management of Public Health Intervention Networks (IMPHI) have been delivered at least annually to participants from member programs like Kenya, Nigeria, Tanzania, Uganda and Zimbabwe with CDC support.

Intra-country networks have also played vital roles in human resource capacity building. The Uganda Immunization Training Program (UITP), for instance, is a collaboration between AFENET, the Uganda Ministry of Health, Uganda National Expanded Programme on Immunisation (UNEPI), WHO and the Task Force for Child Survival. This program, funded by Merck Vaccine Network, has trained over 500 national, midlevel and lower-cadre health staff in Uganda on skills that can improve mother and child survival through better immunization service management.

**Figure 1:**
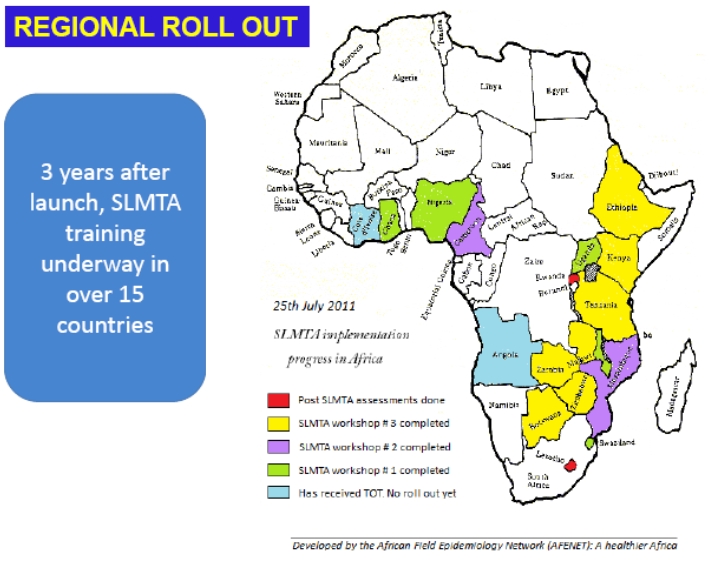
Strengthening Laboratory Management Towards Accreditation (SLMTA) Implementation in Africa

AFENET is a key champion of public health laboratory capacity building and laboratory systems strengthening, a neglected area in many African nations [6]. Through the AFENET-Lab initiatives, AFENET has implemented multiple laboratory strengthening activities in different parts of Africa. These include short-course trainings for frontline and midlevel laboratory personnel which have been conducted in Tanzania, Uganda, and Zimbabwe. Further, AFENET has collaborated with CDC and other partners to pilot a basic laboratory information system (referred to as BLIS) in Uganda and Tanzania with the aim of improving storage and retrieval of laboratory data for better patient management and decision making. In 2010 AFENET-Lab, with the support of the International Laboratory Branch of CDC, began assisting several countries to implement laboratory accreditation activities using the WHO-AFRO Stepwise Laboratory Quality Improvement Process Towards Accreditation, known as SLIPTA. Two years after the launch of the WHO-AFRO Stepwise approach, SLMTA (Strengthening Laboratory Management Towards Accreditation) training activities are underway in over 15 African countries and AFENET has been directly involved in six of these countries namely Angola, Central African Republic, Rwanda, Swaziland, Malawi, Uganda, Tanzania, Zimbabwe. AFENET has also been involved in conducting regional SLMTA Trainer of Trainers (TOT) courses, through provision of technical expertise as SLMTA master trainers. Figure 1 shows progress towards SLMTA implementation in Africa.

## Discussion

AFENET's tripartite working relationship with government technocrats from human health and animal sectors, academicians from partner universities and development partners presents the Network with a vantage point. The Network is able to: leverage resources to support field epidemiology and public health laboratory training and service delivery notably in the area of outbreak investigation and response, disease surveillance; by-pass government bureaucracies that often hinder and frustrate development partners during program implementation; and consolidate efforts of different partners that are channelled through the FELTPs by networking graduates through alumni associations and calling on them to offer technical support in various public health capacities as the need arises. While governments tend to have a long-term perspective on national health issues, the private sector often has short-to-medium term investments in health. AFENET presents a bridging platform allowing for continuity of health interventions at the national and regional level while offering free exit and entry for existing and new partners respectively.

Based on the successes recorded in AFENET's first 5 years, we envision that the Network's membership will continue to expand as new programs are established. The lessons learned over the years such as the need for early involvement of key stakeholders (ministries of health and local universities) in design of new programs and projects being key to project success, will be useful in initiating new programs, and building sustainability frameworks for FETPs and FELTPs in Africa. AFENET will continue to play its role in coordinating, advocacy, and building capacity for epidemic disease preparedness and response.

It is our hope that graduates from member programs will help shape public health policy and practice in Africa. AFENET will continue to play a pivotal role in IDSR implementation and support countries to become IHR compliant. As reported in this paper and elsewhere [24], FELTP alumni take up leadership positions at local, regional and international levels. With a membership that covers over 80% of sub-Saharan Africa, AFENET member FELTPs are destined to not only influence public health decision making by providing public health leaders but to also contribute towards other health systems blocks such as service delivery and leadership/governance.

The health work force in Africa is being strengthened through the 2-year graduate FELTP and 2-week-long in-service short courses of: surveillance and outbreak response, lab management, as well as health leadership and management. Service delivery is enhanced through the trainees’ performance improvement projects, trainees’ participation in outbreak response and other routine public health services during the field component of the training.

AFENET is a key champion of public health laboratory capacity building and laboratory systems strengthening, a neglected area in many African nations. Our role in the roll out of WHO-AFRO's stepwise approach for Strengthening Laboratory Management Towards Accreditation (SLMTA), distribution of outbreak investigation kits to member countries, and in-service training for laboratory personnel, are all testimony to that.

Despite the above successes, AFENET's continued survival and vibrancy is threatened by at least two challenges. The most notable challenge is the need for a reliable stream of funding to sustain the Network's core priorities and maintain the ever increasing number of member programs. As mentioned earlier, FETPs and FELTPs are resource intensive (the average cost of training one person through the 2 year masters’ program is U.S. $ 40,000) [20]. Moreover, most African governments do not have the capacity to fully shoulder all the costs, given competing health needs. This calls for diversification of the funding base, innovation, and design strategies to attract new partners while sustaining existing ones. Health Systems 20/20, with funding from the USAID, helped AFENET develop a 5-year resource mobilization plan and is currently supporting the establishment of a fully fledged business development unit (currently housed under AFENET's Science and Public Affairs unit), diversification of resource mobilization strategies and funding sources.

As the Network continues to expand, harmonization of individual members’ priorities with the collective goals of the network must be considered. Decision making may become difficult given the multiplicity of partners and divergent interests. For example, members may place varying levels of importance on various aspects within their individual program such as academics, research, field service, or improving public health service delivery. Members may prefer to channel resources to address a specific public health priority, or may base priorities on current available funding opportunities. These actions may be inconsistent or incompatible with other members or with the network as a whole. These competing interests must be acknowledged and managed within the context of the network keeping in mind its greater value.

## Conclusion

AFENET presents a versatile networking model that brings together different stakeholders to strengthen African public health systems through field epidemiology and public health laboratory capacity building. Through networking of existing FELTPs and collaboration with development partners, the number of FELTPs has tripled from 4 at inception of AFENET to 12 in 2011, and the number of outbreaks investigated with FELTP involvement has increased. However, there is need for continued support from both development partners and African governments to uphold the highlighted successes and to overcome looming challenges to advancement of field epidemiology implementation in sub-Saharan Africa.

## Abbreviations

AFENET: African Field Epidemiology Network; CDC: Centers for Disease Control and Prevention; EAPHLN: East African Public Health Laboratory Network; EARHN: East African Reproductive Health Network; FELTP: Field Epidemiology and Laboratory Training Program; FETP: Field Epidemiology Training Program; FLU-LAB: Global Influenza Network; GOARN: Global Outbreak Alert and Response Network; IDSR: Integrated Disease Surveillance and Response; IHR: International Health Regulations; SARHN: Southern Africa Reproductive Health Network; TEPHINET: Training Programs in Epidemiology for Public Health Interventions Network; USAID: U.S. Agency for International Development; WARHN: Western African Reproductive Network; WHO: World Health Organisation
